# Multiple voxel pattern analysis shows associations between chronic fatigue syndrome and cortical atrophy

**DOI:** 10.3389/fnins.2025.1535088

**Published:** 2025-03-17

**Authors:** Kang Wu, Yihuai Zou, Yuanyuan Li, Xiaojie Hu, Yahui Wang, Tianzhu Chen, Yuhang Chen, Kuangshi Li

**Affiliations:** ^1^Dongzhimen Hospital, Beijing University of Chinese Medicine, Beijing, China; ^2^Iowa Neuroscience Institute, University of Iowa Carver College of Medicine, Iowa City, IA, United States; ^3^Beijing Tsinghua Changgung Hospital, Tsinghua University, Beijing, China

**Keywords:** chronic fatigue syndrome, cortical atrophy, cortical thickness, MRI, machine learning

## Abstract

Chronic Fatigue Syndrome (CFS) is a disease characterized by unexplained fatigue and impaired cognition for more than 6 months. Recent studies have reported declines in large-scale brain networks’ functional connections among patients with CFS, and these declines correlated with the patients’ symptom severity. However, these reported networks are inconsistent. Brain structure serves as the essential architecture supporting brain functional fluctuations. Investigating structural alterations could provide insights into functional changes in different brain areas and facilitate the clinical diagnosis of CFS. In this study, we recruited 37 patients with CFS and 34 healthy controls to collect their clinical assessments and structural magnetic resonance imaging data. Multiple Voxel Pattern Analysis (MVPA) was employed to recognize chronic fatigue-related brain areas, and cortical thickness was compared between the two groups. By constructing a predictive MVPA classifier with 70% balanced accuracy, we identified five relevant brain areas, including the paracentral cortex, precentral cortex, central cortex, intraparietal cortex, and superior temporal cortex. Subsequently, the results showed that the thickness of these areas had associations with fatigue severity, healthy life status, and pain levels among our subjects. Furthermore, compared to healthy controls, the thickness reduction was observed in patients with CFS. In summary, our study revealed a pathological chronic fatigue pattern for understanding CFS and suggested associations between cortical atrophy and CFS, with the aim of highlighting potential impacts of chronic fatigue. The trial was registered in the Chinese Clinical Trial Registry (ChiCTR2000032577).

## 1 Introduction

Chronic fatigue syndrome (CFS) is a disease characterized by unexplained fatigue lasting for more than 6 months, unrefreshing sleep disorders, and impaired cognition, which could result in low levels of interest in life, work, and social activities, leading to increased physical and mental dysfunctions (Efficace et al., 2013). CFS impacts populations broadly, with an estimated global prevalence of about 0.89% (Lim et al., 2020) and about 53.1% among individuals who have recovered from COVID-19 syndrome (Korompoki et al., 2021). Furthermore, a recent clinical study reported that approximately 91% of people suffering from CFS may not have been formally diagnosed due to insufficient common awareness of the condition (Bateman et al., 2021). That is, nowadays, the potential proportion of patients with CFS may have reached an alarming level and urgently deserves attention. However, our understanding of CFS remains limited, particularly regarding its pathological processes, objective biomarkers, and effective therapies.

CFS was thought to be a metabolic and immune inflammation disease having high levels of blood lactate and urea nitrogen (Osman and Mohamed, 2018; Liu et al., 2018). Results from animal studies indicated that the immune inflammation originated from several dysfunctional brain areas (Zielinski et al., 2019), and these aberrant areas could be divided into two systems. The first is called the broken facilitation system, consisting of the orbitofrontal cortex, prefrontal cortex, and anterior cingulate cortex; the second is called the activated inhibition system, including the insular cortex and posterior cingulate cortex. The interaction between the two systems forms a negative feedback loop that continuously produces fatigue feelings and simultaneously prevents the mind from desiring relaxation (Tanaka et al., 2014). This theoretical model emphasized the crucial role of brain cortex instead of spinal cord or muscle in the processes of CFS, which has been supported by human studies employing non-invasive functional magnetic resonance imaging (fMRI) techniques for CFS. Two task-designed fMRI trials showed that patients with CFS had slower speeds and poorer performance in completing cognitive tests, and brain regions, including dorsal anterior cingulate cortex, occipitoparietal cortex, and cingulate gyrus, were anomalously activated during the cognitive tests, suggesting that these cortices may be recruited to compensate for behavioral insufficiencies (De Lange et al., 2004; Caseras et al., 2008). In contrast to the dynamic enhancement in the task state, patients with CFS tend to exhibit reductions in the resting state. The default mode network (DMN) is the largest brain functional network dominating spontaneous brain activities of attention-demanding and mind-wandering during rest, involving the prefrontal lobe and temporal lobe (Raichle, 2015). Recent fMRI studies reported that patients with CFS had declines in both static and dynamic functional connections (FC) of the DMN, which could significantly identify patients with CFS, and the decline could be moderated as CFS improved (Rayhan and Baraniuk, 2021; Li et al., 2022; Wu et al., 2022). Therefore, this may suggest that the abnormal brain activity pattern—deficient during rest and excessive during activity—could be a pathological process of CFS.

Developing studies consistently demonstrated the FC deficiency in CFS. However, the reported brain regions varied significantly. One study combining arterial spin labeling (ASL) and fMRI approaches showed that patients with CFS had both static and dynamic FCs decreased in the precuneus cortex and right superior parietal lobe, both of which were related to increasing fatigue (Boissoneault et al., 2018). Another study reported weakened static FC in the medial prefrontal cortex, the inferior parietal lobule, and the DMN, with the combination of these regions exhibiting strong diagnostic potential for CFS (Shan et al., 2018). Additionally, a study showed reduced FC in the sensorimotor, frontal, parietal, and temporal cortices, and these reduced FCs were also relevant to increased fatigue severity (Jaeger et al., 2019). The disunity of these results raised concerns about the stability of FC computations and the possibility of treating FC as biomarkers for CFS diagnosis in the future. Practically, FC evaluation relies heavily on computational conditions. First, FC is commonly used for assessing intrinsic properties or interactions among brain functional networks or relationships between prior region seeds and whole brain areas, thus the selection strategy of networks and seeds determines the differences in FC values. Second, pursuing stable and precise FC values requires an fMRI scan lasting more than 30 min (Demeter and Greene, 2024), whereas most current trials are unable to accommodate this requirement. Consequently, a more reliable assessment is recommended to specify altered brain cortices related to CFS.

Assessing brain alterations in terms of brain structure could be more effective than brain function. Brain structure is the fundamental architecture for brain functional fluctuations. Previous studies (Joutsa et al., 2022; Bowren et al., 2022) focusing on brain lesions showed that clinical symptoms can be predicted by the integrity of brain structural voxels, which is evaluated by the differences between broken and normal voxels, revealing a potentially linear and independent relationship of each voxel in structural MRI. However, FC values may not show corresponding changes in the same areas, as they could compensate through coordinated functional networks. Furthermore, increases and decreases in brain structure are meaningful for representing local neural alterations and could be applied directly in clinic settings, whereas FC values are more likely to reflect relative abnormalities comparing other brain regions. In addition, the commonly used preprocessing steps for brain structural images are more unified and reproducible. Structural images involve processing steps of image intensity uniformity correction, tissue segmentation, and surface reconstruction, which can be performed with fewer parameters to define, whereas processing functional images requires first setting up personally preferred parameters for nuisance regressions, bandpass filtering, and smoothing. Given this, investigating brain structural alterations could be a better choice for providing reliable insights into the pathological progress of CFS and facilitating its diagnosis.

Accordingly, in this study, we sought to investigate brain structural alterations in CFS and identify its pathological brain cortices. We first applied multiple voxel pattern analysis (MVPA) to target chronic fatigue-related brain areas. Then, we used a general linear regression model to examine associations between these areas and clinical symptoms. Finally, we compared the thickness of these areas between patients with CFS and healthy controls. The study aims to highlight the potential impacts of CFS.

## 2 Materials and methods

### 2.1 Participants

Due to the high costs of MRI, more than half of previous MRI studies in the domain of machine learning included fewer than 50 subjects (Poldrack et al., 2020). In this trial, we recruited 37 patients with CFS and 34 healthy controls (HC) from Dongzhimen Hospital affiliated to Beijing University of Chinese Medicine from May 2020 to October 2022. The patients with CFS were diagnosed according to 1994 Fukuda CDC criteria (Fukuda et al., 1994) which defines a patient as someone experiencing chronic fatigue lasting over 6 months and this fatigue feeling is not primarily derived from clinical diseases. The HC were age-matched people without chronic fatigue. All subjects were right-handed and aged between 25 and 65, and none had histories of mental disorders or use of psychotropic drugs. Pregnant women, lactating women, women who were menstruating during MRI scanning, and subjects with abnormal brain structure or a body mass index (BMI) > 45, were excluded. This study was approved by the ethics committee of Dongzhimen Hospital affiliated to Beijing University of Chinese Medicine with approval number DZMEC-KY-2019-195 under the Helsinki Declaration of the World Medical Association (2013). All subjects provided their informed and signed consent. The trial was registered in the Chinese Clinical Trial Registry (ChiCTR2000032577).

### 2.2 Clinical assessments

Three clinical questionnaires were applied. The 36-item short form health survey (SF-36) was used to evaluate healthy status; the fatigue scale-14 (FS-14) was used to evaluate fatigue severity; the pain rating index of McGill pain questionnaire (MPQ-PRI) was used to evaluate pain intensity. Patients with CFS tend to have lower SF-36 score, higher FS-14 score, and higher MPQ-PRI score.

### 2.3 Structural MRI data acquisition

Structural MRI data were acquired from an MRI scanner (Siemens Novus, 3.0T, Germany) at the radiology department of Dongzhimen Hospital. Parameters of structural sequence were as follows: repetition time = 1,900 ms, echo time = 2.53 ms, flip angle = 9°, phase encode direction = anterior to posterior, coverage = whole brain including cerebellum, field of view = 250 mm, echo spacing = 7.6 ms, slice thickness = 1.0 mm, and volumes = 176.

### 2.4 Data preprocessing

The structural MRI data were processed through fMRIPrep 20.2.1 (Esteban et al., 2019) by using the option of anatomical image only. Specifically, each subject’s T1-weighted structural image was corrected for intensity non-uniformity. The structural image was then segmented into tissues of gray matter, white matter, and cerebrospinal fluid using OASIS30ANTs template segmented. Further, volume-based spatial normalization was applied to register the structural image to MNI152 space by nonlinear method. Additionally, the corrected structural image was processed by the recon-all command in FreeSurfer 6.0.1 (Dale et al., 1999) to reconstruct brain surface and obtain cortical thicknesses, and the thicknesses were smoothed with a Gaussian kernel of 6 mm^3^. More details can be found at https://fmriprep.org/en/stable/.

### 2.5 Data decoding

Multiple voxel pattern analysis (MVPA) was performed using Python packages Nilearn 0.9.1 (Abraham et al., 2014) and scikit-learn (Pedregosa et al., 2011). First, a random seed was set to enhance reproducibility. Second, subjects’ volume-based gray matter images in standard space were flattened and randomly split into two sets, wherein 35 subjects were in the training set and 36 subjects were in the test set. Then, we constructed a support vector classifier (SVC) with a linear kernel and an L2 penalty in the training set and optimized the hyperparameters by half-split cross-validation. This validation strategy is reported to have the greatest performance in the small sample trials (Valente et al., 2021). Subsequently, we applied the classifier to the test set to evaluate its predictive ability and significance level using half-split 5,000 times permutation tests. Of note, we performed the decoding on the volumetric images because we wanted to include cortical and subcortical areas to achieve higher predictive performance, and we used a balanced accuracy score rather than the actual score because of imbalanced subject numbers in the CFS group and HC group.

**Figure 1:**
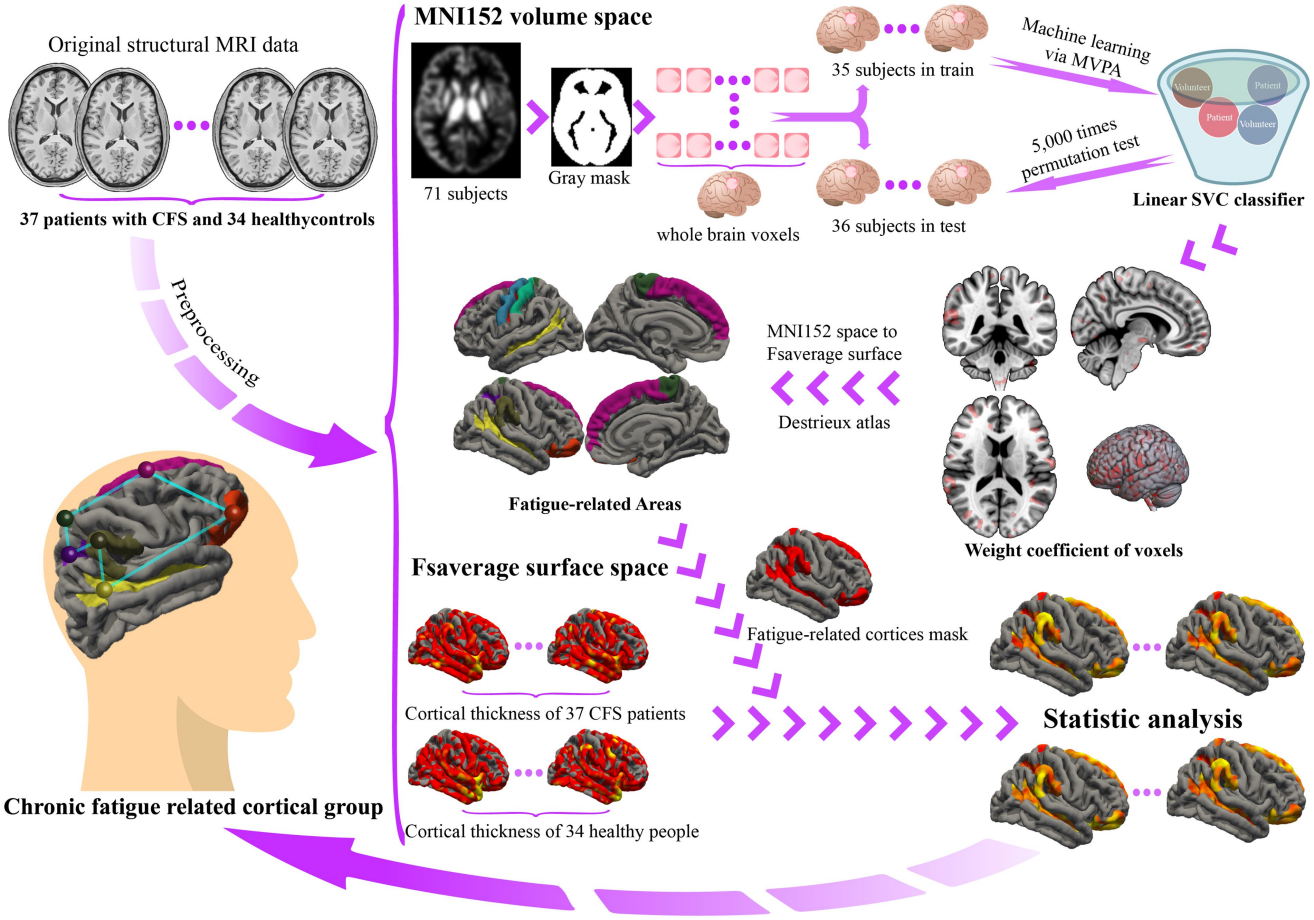
Flow chart of this trial. After preprocessing the original structural MRI data, we constructed the classifier in the volume-based gray matter images. Then, we projected the weighted coefficients of the classifier onto brain surface to obtain accurate brain areas related to CFS, which we named the *chronic fatigue related cortical group*. Consequently, we compared the thickness of these areas between the CFS group and the HC group.

After obtaining the significant classifier, we extracted weighted coefficients from the classifier and projected the coefficients onto fsaverage space using a non-linear normalization tool (Wu et al., 2018). Considering that atlas-based analysis could be more robust and meaningful than cluster-based analysis, we mapped the results to the Destrieux atlas (Destrieux et al., 2010) and computed probabilistic confidence scores for each cortex region. Figure 1 was the flow chart.

### 2.6 Statistical analyses

All statistics were performed in R 4.2.2. Based on the results of Shapiro test, we compared age by Wilcoxon’s test, gender by Chi-squared test, BMI by Student’s *t* test, between the two groups. A general linear regression model with stepwise method was additionally applied to evaluate association size between cortical thicknesses and fatigue scores. The regression model format was $Y_{\mathrm{fatigue}\;\mathrm{scores}}=1+{\mathrm{Thickness}}_{\mathrm{cortex}1}+{\mathrm{Thickness}}_{\mathrm{cortex}2}+\ldots +{\mathrm{Thickness}}_{\mathrm{cortex}12}$ . Finally, the mean value of these cortical thicknesses was compared between the two groups by Wilcoxon’s test. Significant values were corrected by Bonferroni method.

## 3 Results

### 3.1 Clinical assessments comparison

The demographics in Table 1 showed that subjects in the two groups were comparable in terms of age, gender, and BMI. And expected differences showed in scores of SF-36, MPQ-PRI, FS-14, and disease durations between two groups.

**Table 1:** Clinical information comparisons ( $\bar{x\;}$ *±s*).

	Groups		Comparisons	
	CFS	HC	Statistic value	*p* value
Demographics				
*n*	37	34	/	/
Age	35.35 ± 11.98^**^	30.47 ± 11.46^**^	751	0.159
Gender (M/F)	11/26	10/24	0.001	0.977
BMI	22.69 ± 2.52	21.73 ± 2.98	1.474	0.145
CFS durations (month)	58.68 ± 67.98	/	/	/
Clinical assessments				
SF-36	54.40 ± 14.87	82.74 ± 10.79^**^	107	<0.001^*^
MPQ-PRI	7.19 ± 5.01^**^	1.76 ± 1.88^**^	1083.5	<0.001^*^
FS-14	10.24 ± 2.52	4.50 ± 3.75^**^	1,109	<0.001^*^

^**^ represents *p* < 0.05 according to Shapiro test; ^*^ represents *p* < 0.05 according to comparisons between the two groups.

### 3.2 Classifier for recognizing CFS

**Figure 2:**
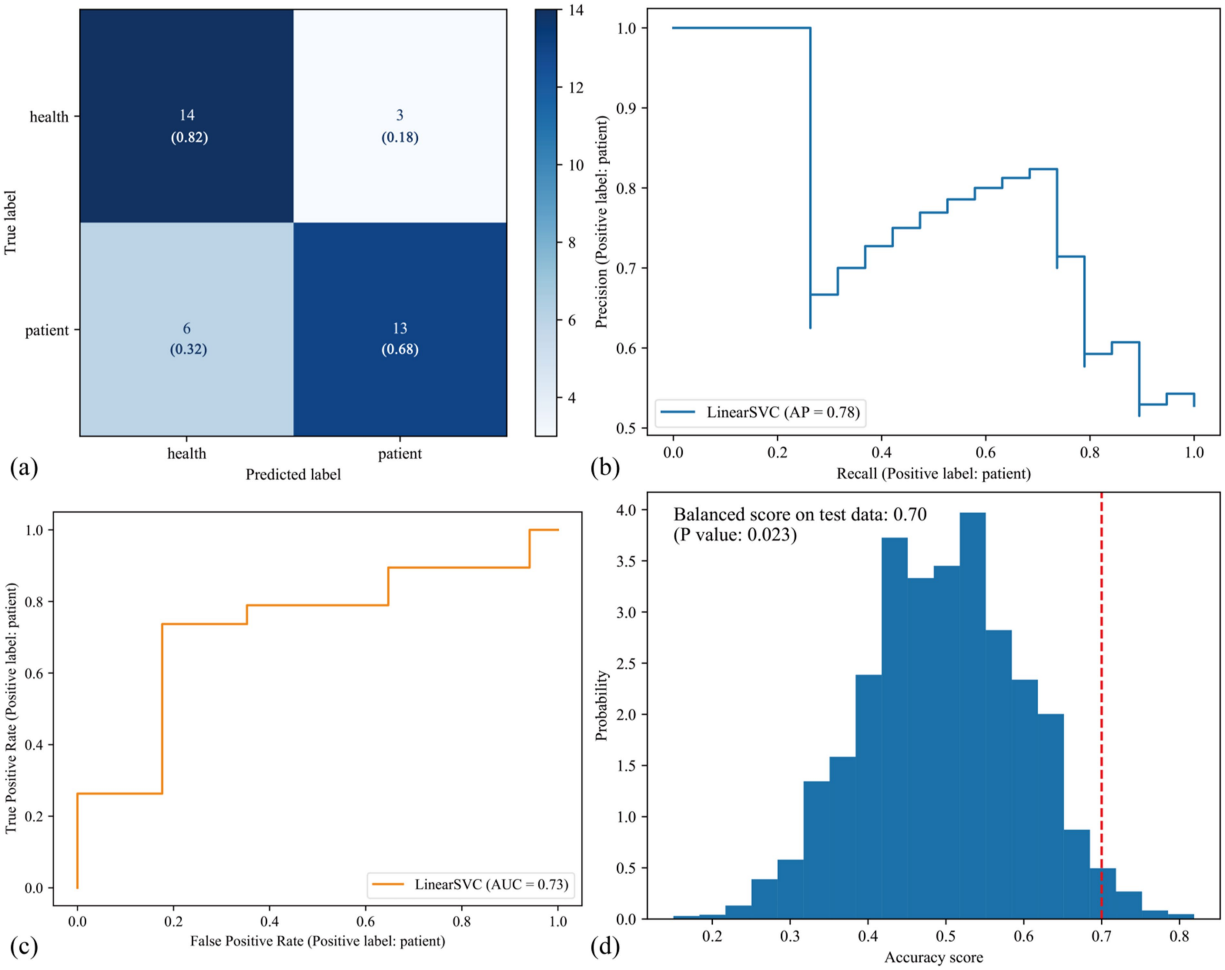
Predictive performances of the classifier in the test set. **(a)** This confusion matrix showed that, among 17 healthy volunteers and 19 patients with CFS in the test set, 27/36 subjects were correctly predicted, resulting in 75% actual accuracy score. **(b)** The precision-recall matrix showed the average precision (AP) of the classifier was 78%. **(c)** The area under the receiver operating characteristic curve (ROC AUC) of the classifier was 73%. The **(a–c)** are three types of measurements to evaluate predictive performances of the classifier. Then we performed half-split 5,000 times permutation tests to determine the significant level of the classifier, and results **(d)** showed that the balanced accuracy score was 70% and the *p* value was 0.023, suggesting that the classifier was statistically significant to recognize CFS.

After training the classifier and optimizing its hyperparameters, we applied the optimized classifier to the test set to evaluate its performances. The confusion matrix showed that 14/17 subjects in the HC group were correctly predicted, resulting in an accuracy score of 82%; 13/19 subjects in the CFS group were correctly predicted, with an accuracy score of 68% (Figure 2a). The precision-recall matrix showed an average precision of 78% (Figure 2b). Additionally, the area under the receiver operating characteristic curve (AUC) of the classifier was 73% (Figure 2c). Permutation tests showed that the balanced accuracy score of the classifier was 70%, with a significant *p* value of 0.023 (Figure 2d). These results indicated that the classifier we constructed can significantly distinguish between the CFS and HC groups.

### 3.3 Chronic fatigue related brain areas

**Figure 3:**
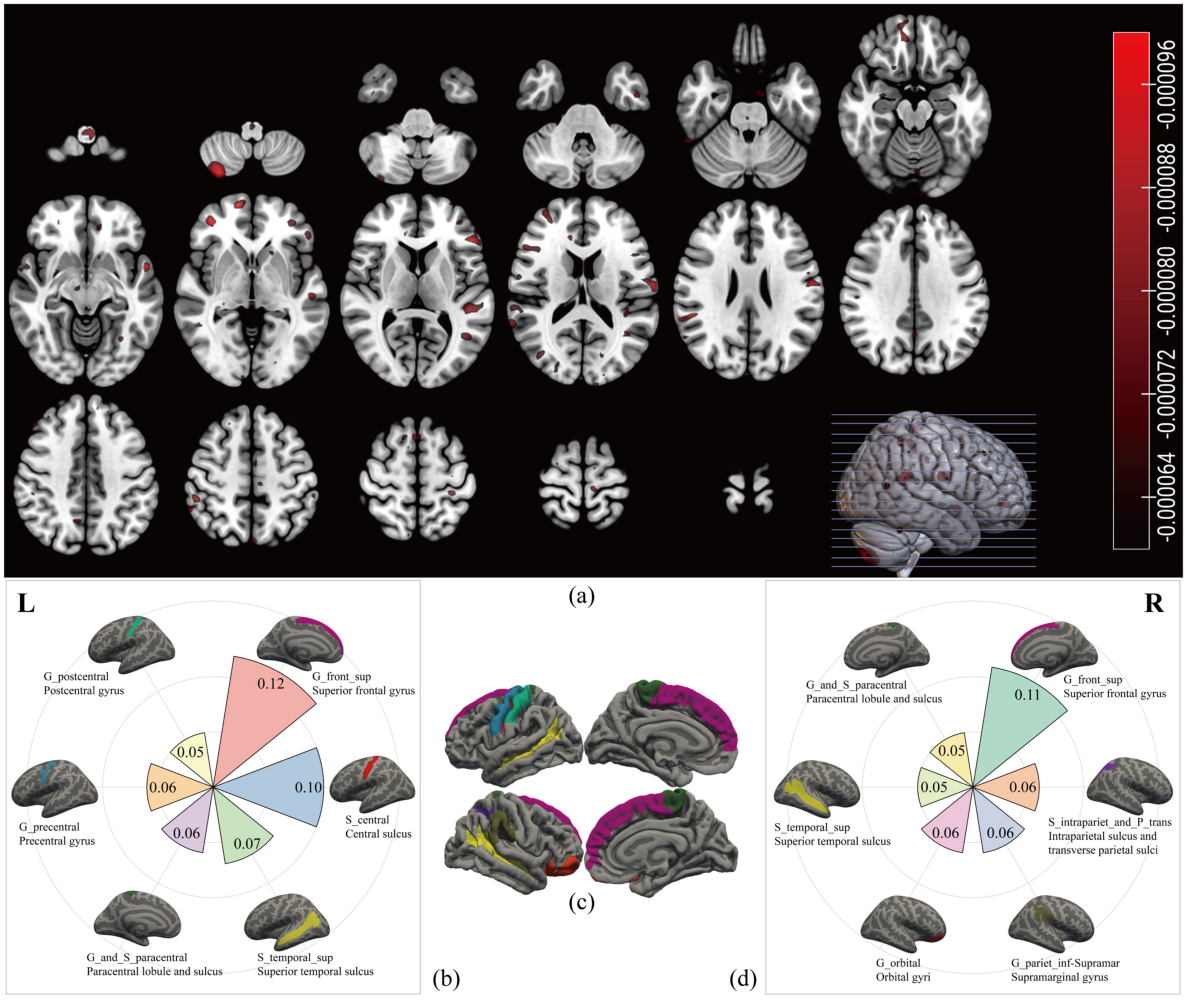
Chronic fatigue related brain areas. We extracted the volume-based weighted coefficients **(a)** from the significant classifier. The red scatters represented the brain areas contributing to the recognition of CFS; most of them were shown on the surface. After projecting the weighted coefficients onto surface space, six regions in the left hemisphere **(b)** and six regions in the right hemisphere **(d)** stood out. The names in the first row with underscores were labels from the Destrieux atlas, and the names in the second row were the full names. Different colours represented different cortices, and the number represented the sum value of the weighted coefficients within each cortex. The **(c)** was the overview map of the 12 cortices related to CFS.

Volume-based weighted coefficients were extracted from the significant classifier, and the voxels contributing to recognition were scattered in the cerebellum and gray matter (Figure 3a). After projecting voxels onto fsaverage template, 12 brain areas stood out, including the superior frontal gyrus, central sulcus, superior temporal sulcus, paracentral lobule and sulcus, precentral gyrus, and postcentral gyrus in the left hemisphere (Figure 3b), and superior frontal gyrus, intraparietal sulcus and transverse parietal sulci, supramarginal gyrus, orbital gyri, superior temporal sulcus, and paracentral lobule and sulcus in the right hemisphere (Figure 3d). To sum up, these cortices related to CFS were primarily in the superior frontal lobe, central lobe, superior temporal sulcus (Figure 3c), occupying 0.85 of the probabilistic confidence score within the classifier.

**Figure 4:**
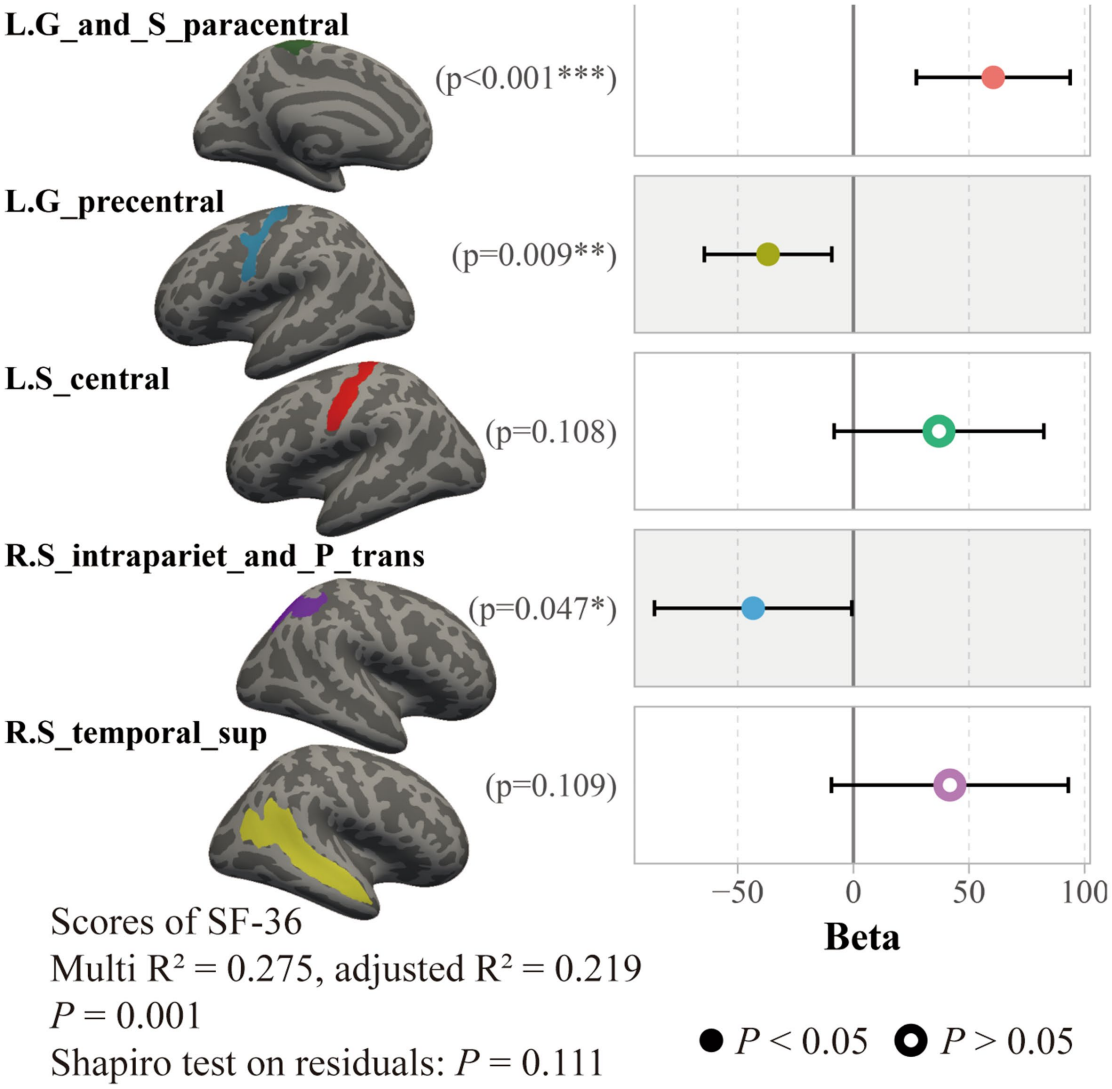
Association between SF-36 scores and cortical thicknesses in all subjects. Results of a general linear regression model showed that thicknesses of these five brain areas had a significant association to SF-36 scores.

**Figure 5:**
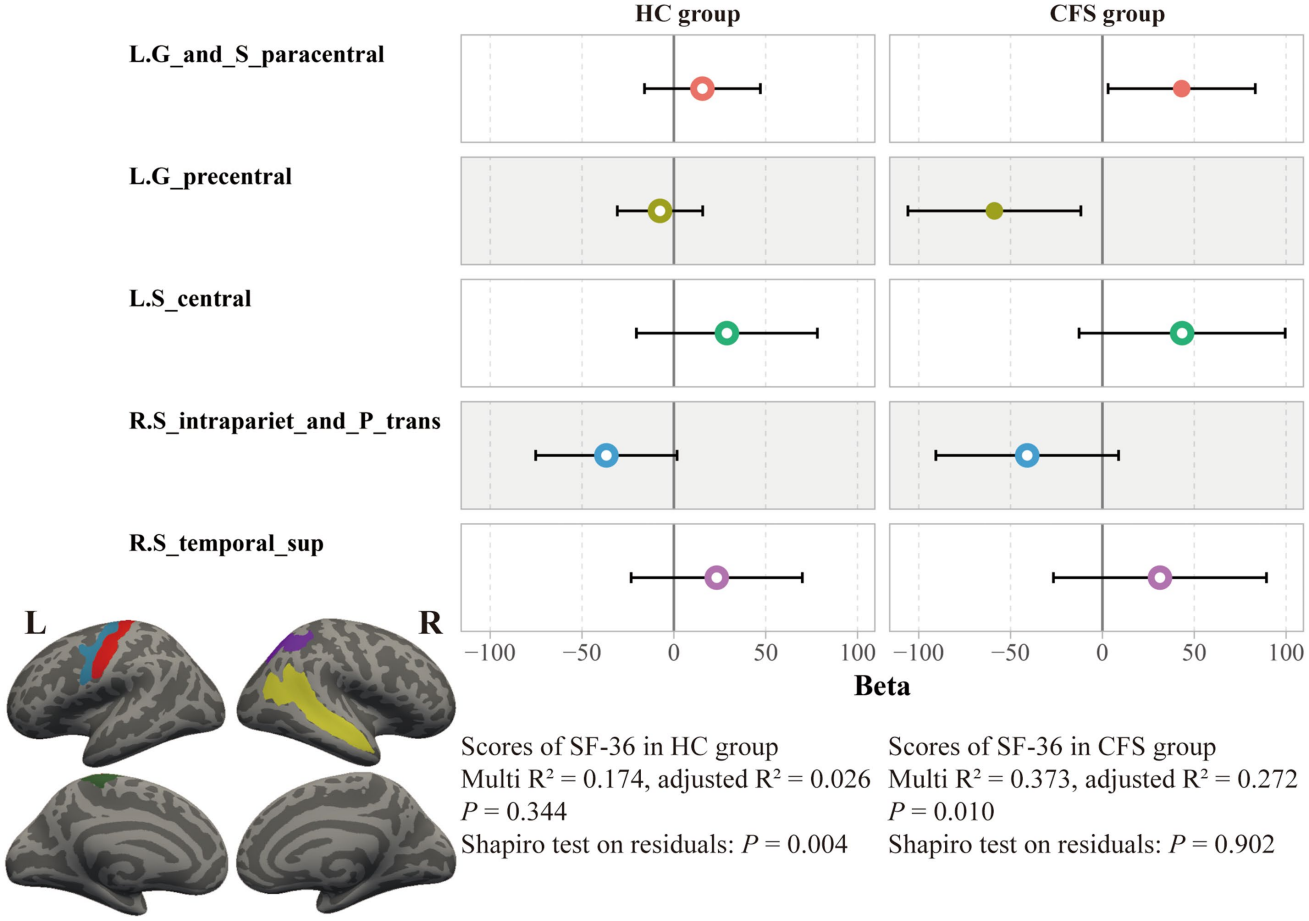
Association between SF-36 scores and cortical thicknesses in the two groups. The association between cortical thicknesses and SF-36 scores was significant only in the CFS group, not the HC group.

By using a general linear regression model between fatigue scores and these cortical thicknesses, five brain areas were retained after pruning by stepwise method, including the left paracentral lobule and sulcus, left precentral gyrus, left central sulcus, right intraparietal sulcus and transverse parietal sulci, and right superior temporal sulcus. These five areas showed significant associations not only with the FS-14 (adjusted *R*^2^ = 0.122, *p* = 0.019, *P* of residuals = 0.120) but also with the SF-36 (adjusted *R*^2^ = 0.219, *p* = 0.001, *P* of residuals = 0.111) and the MPQ-PRI (adjusted *R*^2^ = 0.114, *p* = 0.024, *P* of residuals <0.001). However, only the SF-36 robustly remained the significance when considering multiple corrections (Figure 4). Furthermore, this association was only significant in the CFS group, not the HC group (*p* = 0.010, Figure 5).

### 3.4 Cortical thicknesses comparison

**Figure 6:**
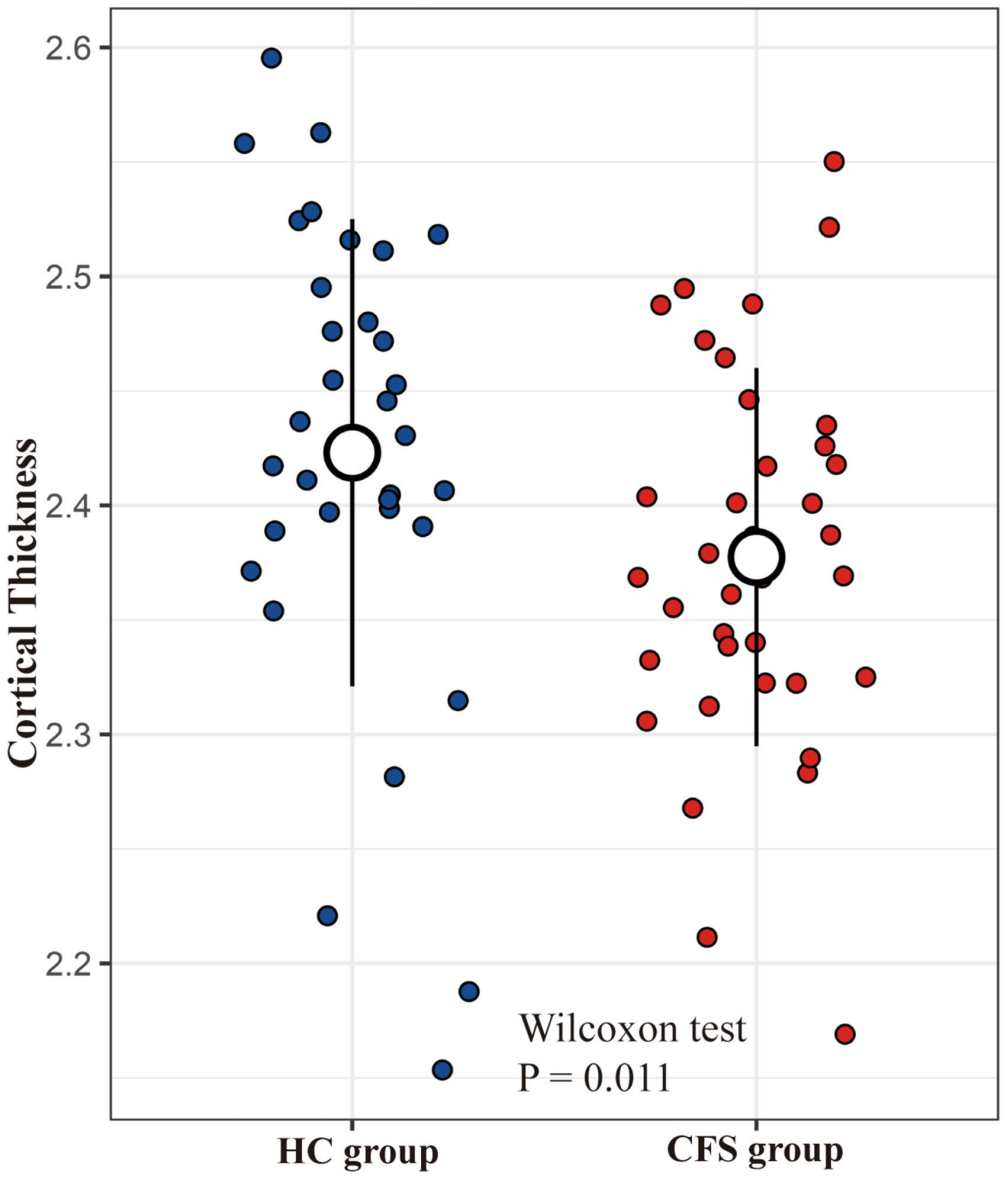
Group comparison of cortical thickness.

We considered the five brain areas to be the pathological areas for CFS, thus we investigated structural alterations of these areas in patients with CFS. Notably, to avoid sensitivity to model coefficients and to enhance robustness and reproducibility, we used the average value of the five brain areas rather than the weighted coefficient value to assess the structural alterations. The average cortical thicknesses of the areas showed a significant difference between the CFS group and HC group (Mean_CFS group_ = 2.37 mm, Mean_HC group_ = 2.43 mm, *p* = 0.011, Figure 6). And this difference was still obvious after eliminating confounding factors of age, gender, and BMI (*p* = 0.034). Additionally, the averaged five cortical thicknesses in the CFS group showed a marginally significant correlation with the CFS duration (Spearman *R* = −0.28, *p* = 0.08).

## 4 Discussion

CFS is reported to have declines in functional connections, but the specific brain areas involved are inconsistent. Brain structure supports functional fluctuations, thus investigating structural alterations may contribute more effectively to clinical diagnosis of CFS and provide insights into functional declines. In this study, we constructed a predictive classifier to significantly recognize patients with CFS from healthy individuals. Five brain areas were identified by the classifier, and these areas showed significant explanation ratios to clinical assessments in patients with CFS. Subsequently, we observed decreased cortical thickness in patients with CFS compared to healthy controls. These results suggest that the five brain areas we found could be pathological regions for CFS, and cortical atrophy may be related to long-term fatigue.

To our knowledge, this is the first study employing the MVPA method to examine structural alterations for CFS. Analysing all voxels of structural images is a powerful strategy to reveal potential pathological patterns for diseases, which is based on the simple assumption that neural functions or deficits originate from insufficiencies in its structural architecture, and this strategy has been applied for a long time (Mah et al., 2014). In this study, our classifier constructed by all voxels showed the capacity for recognizing CFS in terms of many predictive indicators, resulting in a stable accuracy score of 70%. Since there has not been same research, our accuracy score could be treated as a comparable baseline for future structural models for CFS. As shown by our classifier, scattered voxels with high weighted coefficients were in the brain surface, occupying a large proportion of weights within the model, which showed consistent with the previous study conducting dysfunctional areas of CFS in the brain cortices (Tanaka et al., 2014). To accurately target the CFS related brain areas, we then projected voxels to the brain surface and classified them according to an anatomic atlas.

As revealed by our classifier, five brain areas were apparently relevant to CFS, including the left paracentral lobule and sulcus, left precentral gyrus, left central sulcus, right intraparietal sulcus and transverse parietal sulci, and right superior temporal sulcus. Our results showed these five areas have significant explanation ratios to scores of SF-36, FS-14, and MPQ-PRI (Figures 4, 5). These brain areas are meaningful. The paracentral lobule and sulcus, precentral gyrus, and central sulcus are near the motor cortex and related to our somato-cognitive actions (Gordon et al., 2023). Previous studies showed that the reduced thickness in the paracentral lobule cortex could be a potential symbol for individuals at high risk of mental illnesses (Sasabayashi et al., 2021), while the reduced thickness in the precentral sulcus might lead to higher rates of suicidal tendencies and mood dysfunctions (Papmeyer et al., 2015). Additionally, the reduced thicknesses in the paracentral and precentral gyrus may make individuals vulnerable to psychosis (Takayanagi et al., 2020). The superior temporal cortex is the primary region for multi-sensory integration with somatosensory, auditory, and visual stimulation, cooperating with motor-related cortices to perform cognitive functions and tasks. If reduced thickness is observed in this region, it would result in anxiety and depressive symptoms with cognitive impairments, emotional symptoms, and poor sleep quality (Wang et al., 2021). These related results indicated the five brain areas we found could be critical regions for regulating mental dysfunction in CFS, suggesting close connections between CFS and psychiatric disorders. In fact, several studies have demonstrated that CFS and depression have strong co-occurrences and shared pathological pathways (Chaves-Filho et al., 2019; Maes, 2011), and about 30% of patients with CFS experience depressive symptoms (Maes, 2009; Loades et al., 2016; Loades et al., 2021). However, whether CFS can be defined as a type of psychiatric disorder requires further study. For these five brain areas, we named them ‘*the chronic fatigue cortical group*’, which could facilitate our understanding of chronic fatigue. In the future, studies could focus on this cortical group to investigate the dose-effect relationship between the integrity of this pathological group and fatigue severity to promote the clinical diagnosis of CFS.

Compared to the healthy group, the five brain areas showed a significant thickness decline in the CFS group, and the decline was still obvious after excluding demographic confounds. Further, thicknesses of the five brain areas showed a moderate anticorrelation with CFS durations, although the anticorrelation was not statistically significant due to our small sample size. These results indicated that limited cortical atrophy may occur in individuals affected by fatigue for a long time, although we cannot specify whether the durations and the atrophy are causally related. Previous literature includes two studies focusing on potentially cortical thickness declines in patients with CFS, but each drew a different conclusion. One study conducted a whole-brain analysis on CFS, reporting thickness reductions in the caudal middle frontal gyrus and precuneus (Thapaliya et al., 2022), while another study concluded no global grey matter thickness differences between patients with CFS and healthy individuals (van der Schaaf et al., 2017). Our results emphasized the relationship between cortical atrophy and chronic fatigue, further specifying that the atrophy could be limited to five brain areas related to CFS. Compared to the previous two studies, our trial, based on advanced machine learning method, provided more precise pathological regions. Additionally, our trial exhibited relationships between the cortical atrophy and fatigue severity, healthy status, pain severity, and symptom duration in patients with CFS. However, further longitudinal studies are required to investigate potential causality between cortical atrophy and chronic fatigue.

One interesting result of this study that drew our attention was that the healthy status scores, rather than the fatigue severity scores, exhibited the highest explanatory ratios and robust significance. We identified two possible reasons. First, the assessments of fatigue severity we used only had 14 questions, fewer than the healthy status questionnaire with 36 questions. More items and scoring ranks may provide higher resolution for better model fitting. Second, and more importantly, fatigue severity evaluated by FS-14 may not precisely reflect chronic fatigue and acute fatigue, because healthy volunteers could have acute or broad fatigues derived from work stresses and social competition. From Table 1, the fatigue severities of our HC group were 4.50 ± 3.75, and seven subjects in the group were assessed as having moderate fatigue severities (score > 7) on the day they were recruited, even though they reported being healthy and having no history of long-term fatigue. This is reasonable and cannot be simply attributed to the possible loose criteria for healthy volunteers in our trial. In practice, acute fatigue is often considered an early stage of chronic fatigue and is broadly present. For instance, occupational fatigue is one of the common sources of acute fatigue, such as 12-h shift nurses (Chen et al., 2014), car drivers, and aircraft pilots (Hu and Lodewijks, 2020), while temporary inflammation is another common source of acute fatigue in healthy subjects (Lasselin et al., 2020). Unlike chronic fatigue, healthy individuals with acute fatigue can recover by themselves through sufficient relaxation, although acute fatigue may temporarily recur when fatigue-inducing factors arise. Compared to the FS-14 questionnaire, the SF-36 could evaluate lifestyle changes in subjects over 1 month, which may potentially provide details to help distinguish between acute and chronic fatigue. Hence, we thought that unless a new questionnaire assessing fatigue in multiple dimensions and time points is developed, the SF-36 could possibly be considered a better outcome for studying CFS in some cases under the current situation. Future studies could focus more on the distinct pathological patterns of acute and chronic fatigue.

The limitations of this study include that our sample size could potentially restrict the reliability of our results, although the number of participants in our study exceeded most previous similar studies, several conclusions from this study may require a large, multi-center study with a more diverse patient pool to enhance statistical power and improve reliability. Second, our study focused on the brain surface and ignored subcortical regions, which means the pathological patterns we revealed could be incomplete, such as the critical role of amygdala in CFS. Third, several conclusions from this cross-sectional study could be influenced by explanatory bias and observer effects. Finally, anxiety and depressive scores were not included in the analyses of this study, although we ensured that the recruited subjects had no histories of mental disorders or psychotropic drug use. Our future study will investigate these conclusions in patients with depression and anxiety.

## 5 Conclusion

In conclusion, we constructed a classifier to recognize CFS and identified pathological patterns for chronic fatigue. Five brain areas, including the left paracentral lobule and sulcus, left precentral gyrus, left central sulcus, right intraparietal sulcus and transverse parietal sulci, and right superior temporal sulcus, were associated to clinical symptoms of CFS, and patients with CFS exhibited cortical atrophy in these five areas. Our study could promote the understanding of CFS and kindly remind potential impacts of chronic fatigue. Besides, the pathological patterns we revealed could contribute to clinical diagnosis of CFS.

## Data Availability

The datasets presented in this study can be found in online repositories. The names of the repository/repositories and accession number(s) can be found at: https://github.com/Clancy-wu/PaperScripts/tree/main/mvpa-vbm-analysis-CFS-baseline.
